# Metalloprotease ADAM9 cleaves ephrin-B ligands and differentially regulates Wnt and mTOR signaling downstream of Akt kinase in colorectal cancer cells

**DOI:** 10.1016/j.jbc.2022.102225

**Published:** 2022-07-01

**Authors:** Pathirennehelage Chandrasekera, Mark Perfetto, Congyu Lu, Minghui Zhuo, Harinath Bahudhanapati, Jiejing Li, Wei-Chih Chen, Pallavi Kulkarni, Laura Christian, Jun Liu, Yvette Y. Yien, Chundong Yu, Shuo Wei

**Affiliations:** 1 Department of Biological Sciences, University of Delaware, Newark, Delaware, USA; 2 Department of Biology, West Virginia University, Morgantown, West Virginia, USA; 3 Pittsburgh Heart, Lung and Blood Vascular Medicine Institute and Department of Medicine, University of Pittsburgh, Pittsburgh, Pennsylvania, USA; 4 State Key Laboratory of Cellular Stress Biology, Innovation Center for Cell Biology, School of Life Sciences, Xiamen University, Xiamen, China; 5 Department of Clinical Laboratory, The Affiliated Hospital of KMUST, Medical School, Kunming University of Science and Technology, Kunming, China; 6 Department of Biochemistry and Cancer Institute, West Virginia University School of Medicine, Morgantown, West Virginia, USA

**Keywords:** ADAM, Akt/PKB, colorectal cancer, ephrin, mTOR, Wnt signaling, ADAM, A Disintegrin and Metalloprotease, APC, adenomatous polyposis coli, cDNA, complementary DNA, CRC, colorectal cancer, EGFR, epidermal growth factor receptor, FBS, fetal bovine serum, HRP, horseradish peroxidase, KD, knockdown, PP2A, protein phosphatase 2A, pro-HB-EGF, pro-heparin binding-EGF, RTK, receptor tyrosine kinase, RT-qPCR, quantitative RT-PCR, TSC2, tuberous sclerosis complex 2

## Abstract

Ephrin-B signaling has been implicated in many normal and pathological processes, including neural crest development and tumor metastasis. We showed previously that proteolysis of ephrin-B ligands by the disintegrin metalloprotease ADAM13 is necessary for canonical Wnt signal activation and neural crest induction in *Xenopus*, but it was unclear if these mechanisms are conserved in mammals. Here, we report that mammalian ADAM9 cleaves ephrin-B1 and ephrin-B2 and can substitute for *Xenopus* ADAM13 to induce the neural crest. We found that *ADAM9* expression is elevated in human colorectal cancer (CRC) tissues and that knockdown (KD) of ADAM9 inhibits the migration and invasion of SW620 and HCT116 CRC cells by reducing the activity of Akt kinase, which is antagonized by ephrin-Bs. Akt is a signaling node that activates multiple downstream pathways, including the Wnt and mTOR pathways, both of which can promote CRC cell migration/invasion. Surprisingly, we also found that KD of ADAM9 downregulates Wnt signaling but has negligible effects on mTOR signaling in SW620 cells; in contrast, mTOR activity is suppressed while Wnt signaling remains unaffected by ADAM9 KD in HCT116 cells. These results suggest that mammalian ADAM9 cleaves ephrin-Bs to derepress Akt and promote CRC migration and invasion; however, the signaling pathways downstream of Akt are differentially regulated by ADAM9 in different CRC cell lines, reflecting the heterogeneity of CRC cells in responding to manipulations of upstream Akt regulators.

The Eph receptors are the largest class of receptor tyrosine kinases (RTKs) and key regulators of cell adhesion, migration, and sorting. These receptors can be divided into two subclasses based on their cognate ligands: EphA receptors, which preferentially bind the glycosylphosphatidylinositol-anchored ephrin-A ligands, and EphB receptors, which preferentially bind the transmembrane ephrin-B ligands (1, 2). Unlike the prototypical RTK pathways, which activate downstream Ras-Erk and PI3K-Akt signaling, activation of Eph receptors by the ephrin ligands (“forward” signaling) often results in inhibited Ras-Erk and Akt activities (3). In addition, Eph–ephrin interactions can also stimulate “reverse” signaling in ligand-expressing cells (1, 2, 3). Eph–ephrin signaling has been widely implicated in cancer and can either promote or suppress tumorigenesis, depending on the context (4). In colorectal cancer (CRC), forward ephrin-B signaling suppresses tumor progression including metastasis, and EphB expression is frequently silenced at adenoma-carcinoma transition (5, 6).

Protein ectodomain shedding, often mediated by members of A Disintegrin and Metalloprotease (ADAM) family, is a key mechanism for regulating cell signaling (7). ADAMs are multidomain type I transmembrane proteins consisting of extracellular pro-, metalloprotease, disintegrin, and cysteine-rich domains (8, 9). More than half of the ADAMs contain a conserved zinc-binding motif in the metalloprotease domain and can cleave (“shed”) the ectodomain of cell-surface substrates (8, 9). Several ADAMs are enriched in tumors and have important roles in various aspects of tumor progression, such as growth, chemoresistance, immune evasion, angiogenesis, and metastasis (9, 10).

Eph–ephrin interactions can be regulated by receptor or ligand ectodomain shedding, typically resulting in loss or termination of signaling (11, 12). One interesting example is the shedding of EphA2 receptor by MMP14, a metalloprotease distantly related to ADAMs, in tumor cells. This proteolytic event abrogates tumor-suppressive forward signaling and promotes oncogenic ligand-independent signaling, thereby converting EphA2 from a tumor suppressor to an oncoprotein (13). Shedding also occurs on ephrin ligands, and the best characterized ephrin sheddase is ADAM10, which can cleave ephrin-A2, -A5 and -B2 (14, 15, 16, 17). Additionally, we have shown that shedding of ephrin-B1 and -B2 by ADAM13 attenuates forward ephrin-B signaling, which antagonizes the canonical Wnt pathway, to induce the neural crest and eye field in *Xenopus* embryos (18, 19). However, it remained unclear how ephrin-B shedding affects Wnt signaling, and whether these shedding events are conserved in mammals.

In the present study, we found that mammalian ADAM9 cleaves ephrin-B1 and -B2 and can substitute for *Xenopus* ADAM13 in neural crest induction. *ADAM9* mRNA is elevated in human CRC tissues and facilitates CRC cell migration and invasion *in vitro via* Akt activation. Our data further reveal that the Wnt and mTOR pathways downstream of Akt show different responses to ADAM9 knockdown (KD) in different CRC cell lines, likely due to the presence of different mutations in these cells.

## Results

### ADAM9 cleaves ephrin-B1 and -B2 to downregulate forward ephrin-B signaling and can functionally substitute for *Xenopus* ADAM13

**Figure 1:**
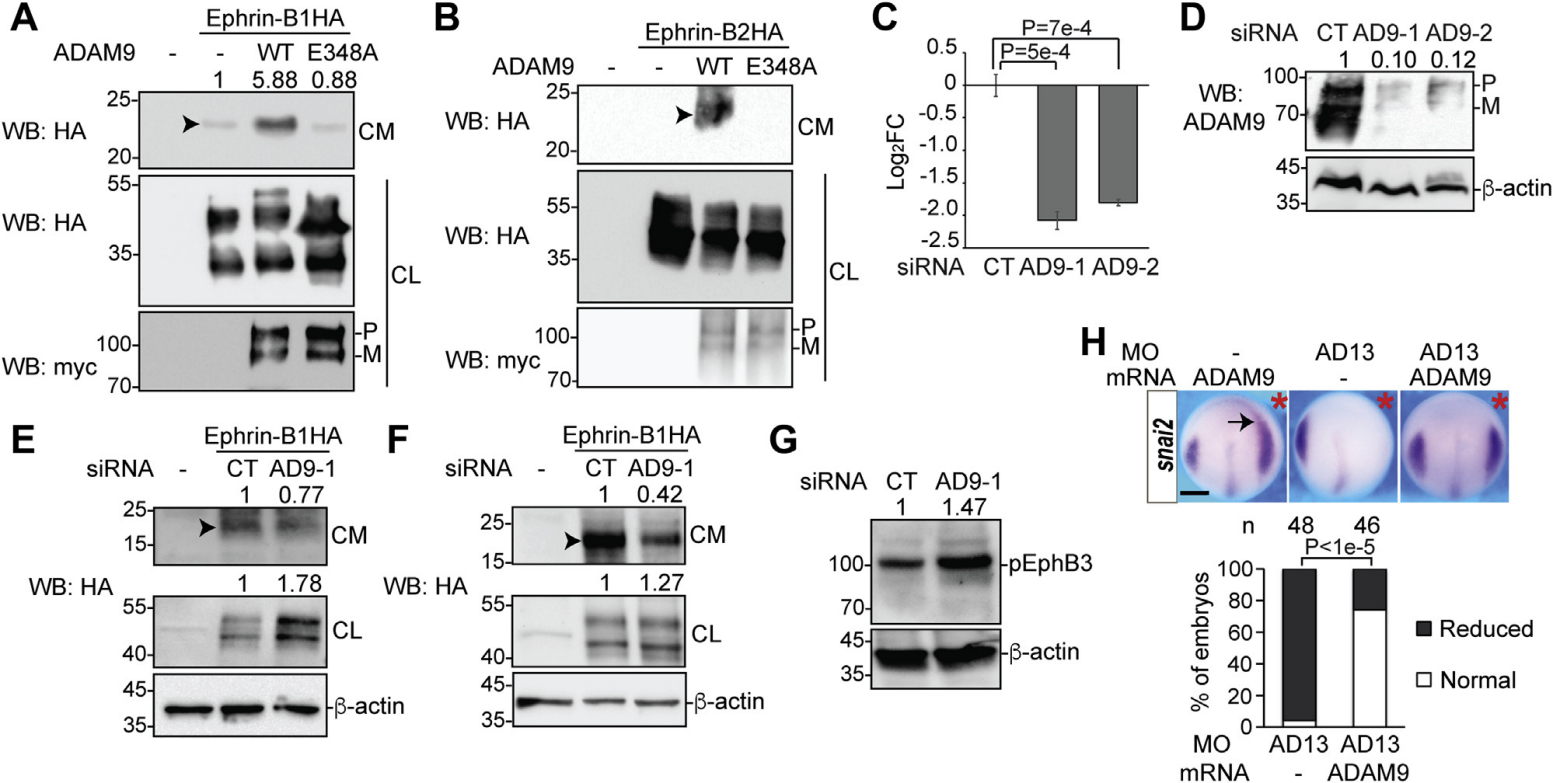
**ADAM9 cleaves ephrin-B1 and -B2 and can substitute for *Xenopus* ADAM13 in neural crest induction.***A* and *B*, HEK293T cells were transfected with plasmids encoding N-terminally HA-tagged ephrin-B1 *(A)* or -B2 (*B*) and C-terminally myc-tagged WT ADAM9 or the E348A mutant, and Western blotting was carried out using the indicated antibodies. Arrowheads point to the shed ectodomain of ephrin-Bs. *C* and *D*, HCT116 cells were transfected with the indicated siRNA, and RT-qPCR (*C*) and Western blotting (*D*) were carried out for ADAM9 mRNA and protein, respectively. Unpaired *t* tests were performed for log_2_FC of mRNA levels obtained for three biological replicates in (*C*), and error bars represent SEM. *E*-*G*, HCT116 (*E* and *G*) or SW620 (*F*) cells were transfected with the indicated siRNA and plasmid, and Western blotting was carried out using an anti-HA (*E* and *F*) or phospho-EphB3 (*G*) antibody. *H*, two-cell stage *Xenopus tropicalis* embryos were injected in one blastomere with the indicated MO and mRNA, allowed to develop to stage ∼12.5, and processed for *in situ* hybridization for *snai2*. χ^2^ test was performed for the percentage of normal embryos (n indicates total number of embryos examined). *Red asterisks* denote the injected side, and *arrow* indicates the expansion of *snai2* expression domain. The scale bar represents 250 μm. CL, cell lysates; CM, conditioned media; CT, control; FC, fold change; M, mature form; P, pro-form (same below). ADAM, A Disintegrin and Metalloprotease; RT-qPCR, quantitative RT-PCR; MO, morpholino.

In a candidate screen to search for mammalian metalloproteases responsible for shedding key cell-surface proteins involved in Wnt signaling, we identified ADAM9 as a main sheddase for mouse ephrin-B1 and -B2 when coexpressed in HEK293T cells (Bahudhanapati *et al.*, unpublished data). Since there is no good antibody for the ectodomain of either ephrin, we overexpressed both ephrins with an N-terminal HA tag. Western blotting detected a fragment of HA-tagged ephrin-B1 ectodomain shed into the conditioned media, which was enhanced when myc-tagged WT mouse ADAM9 but not the protease-dead E348A mutant was coexpressed (Fig. 1*A*). A similar cleavage product was detected for ephrin-B2, although coexpression of WT ADAM9 and longer exposure were needed (Fig. 1*B*). The shed ectodomain of both ephrins are ∼22 kDa, similar to the size of *Xenopus* ephrin-B2 shed by ADAM13 (18), indicating that mouse ADAM9 and *Xenopus* ADAM13 cleave at the same region of ephrin-B ectodomain. Because ephrin-B1 appeared to be cleaved by endogenous ADAM9-like activity, we focused on ephrin-B1 for the rest of this study. To test if endogenous ADAM9 is responsible for cleaving ephrin-B1, we used two siRNAs to knock down ADAM9 in the CRC cell line HCT116, which expresses relatively high levels of ADAM9. Quantitative RT-PCR (RT-qPCR) results show that both siRNAs, AD9-1 and AD9-2, caused 70 to 80% reduction in ADAM9 mRNA, and Western blotting confirms that the endogenous ADAM9 protein was also markedly reduced (Fig. 1, *C* and *D*). Transfection with either siRNA decreased the ephrin-B1 ectodomain shed into the conditioned media and increased the full-length ephrin-B1 in the cell lysate (Fig. 1*E* and S1), pointing to ADAM9 as the main protease responsible for shedding ephrin-B1 in HCT116 cells. Similar effects were observed when ADAM9 was knocked down in SW620, another CRC cell line (Fig. 1*F*). It has been shown that ephrin-B1 and EphB3 are the only ephrin-B ligand and functional EphB receptor, respectively, detectable in HCT116 cells (20). KD of ADAM9 in these cells led to an increase in activated (phosphorylated) EphB3 (Fig. 1*G*), indicating an upregulation of forward ephrin-B signaling, possibly by blocking ligand cleavage. We further tested if mouse ADAM9 is the mammalian functional equivalent of *Xenopus* ADAM13. Like *Xenopus* ADAM13 (18, 21), ectopic mouse ADAM9 expanded the expression domain of *snai2*, a neural crest marker and Wnt target (22), and rescued the decrease of *snai2* caused by ADAM13 morpholino in *Xenopus tropicalis* embryos (Fig. 1*H*). Thus, mammalian ADAM9 can functionally substitute for *Xenopus* ADAM13 to cleave ephrin-Bs and induce the neural crest.

### *ADAM9* is upregulated in human CRC tissues and promotes CRC cell migration and invasion *in vitro*

**Figure 2:**
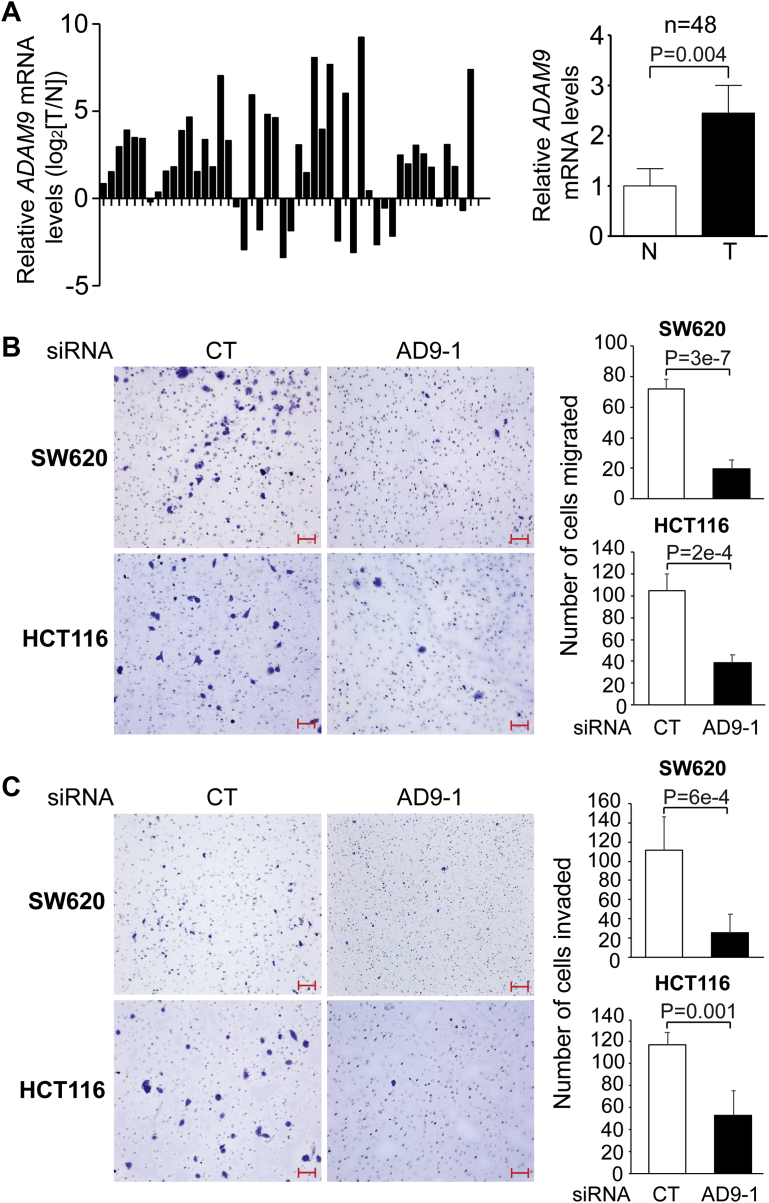
***ADAM9* mRNA is upregulated in human CRC samples and promotes CRC cell migration and invasion *in vitro*.***A*, comparison of *ADAM9* transcripts in CRC tissues (T) with matched adjacent normal tissues (N) from individual patients, as measured by RT-qPCR analyses (left). Results of 48 samples are summarized on the right, and Wilcoxon matched-pairs signed rank test was performed. *B* and *C*, SW620 or HCT116 cells were transfected with the indicated siRNA, and transwell migration (*B*) and invasion (*C*) assays were carried out as described in Experimental procedures. Results of three biological replicates for each treatment are summarized on the right, and unpaired *t* test was performed. Error bars represent SD. The scale bars represent 100 μm. ADAM, A Disintegrin and Metalloprotease; CRC, colorectal cancer; RT-qPCR, quantitative RT-PCR.

Since there is no established mammalian model for neural crest induction (23, 24), we decided to investigate the function of ADAM9 in other physiological contexts. Forward ephrin-B signaling is a tumor suppressor pathway that prevents CRC progression (5, 25), whereas ADAM9 is highly expressed in multiple types of solid tumors, and the expression levels often correlate with tumor progression (26). However, little is known about the endogenous expression and function of this protease in CRC. We therefore carried out RT-qPCR to determine the expression of *ADAM9* in human CRC tissues. As shown in Figure 2*A*, *ADAM9* mRNA was significantly upregulated in CRC tissues as compared with adjacent nontumorous tissues taken from the same patients, suggesting a role for this protease in CRC carcinogenesis and/or tumor progression. Indeed, KD of ADAM9 using the siRNA AD9-1 inhibited the migration and invasion of both SW620 and HCT116 cells, in transwell assays (Fig. 2, *B* and *C*). Similar inhibition was obtained in both cell lines using the siRNA AD9-2 (Fig. S2), confirming that these phenotypes are specific for ADAM9 KD.

### KD of ADAM9 suppresses Wnt activity in SW620 but not HCT116 cells

**Figure 3:**
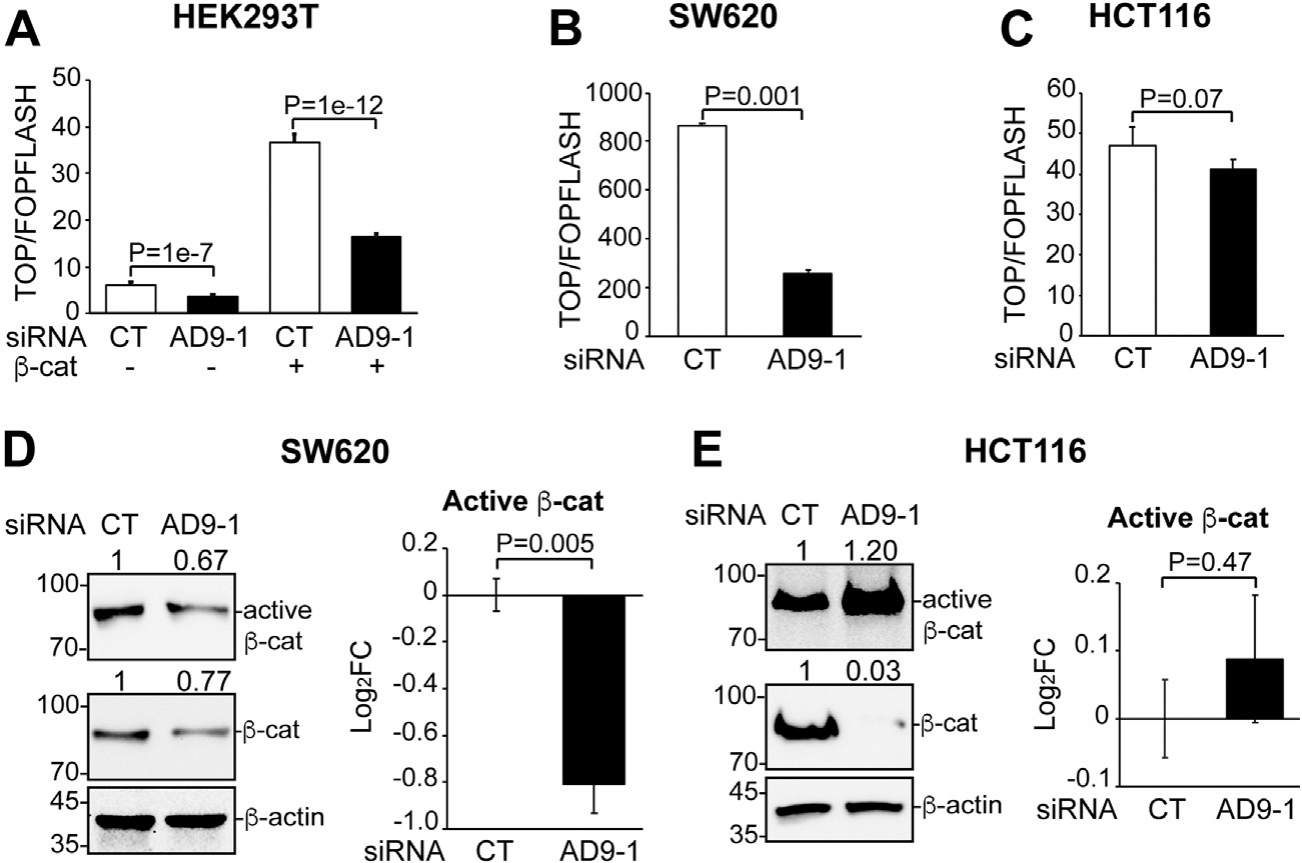
**KD of ADAM9 inhibits Wnt activity in HEK293T and SW620 but not HCT116 cells.** Cells were transfected with the indicated siRNA and plasmid. *A-C*, TOP/FOPFLASH luciferase reporter assays were carried out in triplicate with the indicated cell lines, and unpaired *t* tests were performed to compare Wnt activity. Error bars represent SD. *D* and *E*, Western blotting was carried out for cell lysates with the antibodies for total and active β-catenin, as indicated, in SW620 (*D*) or HCT116 (*E*) cells. Western blotting was repeated in triplicate to confirm the effects of ADAM9 KD on active β-catenin (see Fig. S3 for images of blots); unpaired *t* tests were performed for log_2_FC of protein levels and summarized in graphs (error bars represent SEM). ADAM, A Disintegrin and Metalloprotease; KD, knockdown.

We previously attributed the ability of *Xenopus* ADAM13 to induce the neural crest to the proteolysis of ephrin-Bs and downregulation of forward ephrin-B signaling, which antagonize canonical Wnt signaling, a major signaling pathway that induces the neural crest (18, 19). KD of ADAM13 in *X. tropicalis* embryos inhibited endogenous Wnt activity at the neural plate border, where the neural crest is normally induced (18, 21). Likewise, KD of ADAM9 in HEK293T cells also reduced both the endogenous and ectopic β-catenin–induced Wnt activity in the TOP/FOPFLASH luciferase reporter assays (Fig. 3*A*). A similar reduction was observed in SW620 cells (Fig. 3*B*). Because Wnt signaling can facilitate CRC migration and invasion (27), this result may explain the reduced migration/invasion of SW620 cells upon ADAM9 KD. Surprisingly, we were unable to detect a significant effect of ADAM9 KD on Wnt-mediated transcriptional activity in HCT116 cells using the TOP/FOPFLASH assays (Fig. 3*C*). The cellular levels of β-catenin are usually kept in check by the kinase GSK3, which forms a complex with the scaffold proteins adenomatous polyposis coli (APC) and Axin to phosphorylate β-catenin at Thr41, Ser37, and Ser33 residues, leading to the ubiquitination and degradation of β-catenin. Binding of Wnt ligands to cell-surface Frizzled receptors and coreceptors causes inhibition and dissociation of the β-catenin destruction complex, and the stabilized β-catenin can translocate into the nucleus, where it functions as a transcriptional coactivator (28, 29). However, Wnt-mediated transcriptional activity does not always correlate with total β-catenin; instead, it correlates well with an “active” form of β-catenin, which is dephosphorylated at Thr41 and Ser37 by an unknown phosphatase and is mostly localized in the nucleus (30). Our Western blotting analyses show that both total and active (nonphosphorylated) β-catenin levels were reduced in SW620 cells upon ADAM9 KD (Figs. 3*D* and S3*A*). In stark contrast, no reduction in active β-catenin was detected in HCT116 cells, despite a decrease of total β-catenin (Figs. 3*E* and S3*B*). The lack of reduction in active β-catenin provides an explanation for the lack of response of Wnt-mediated transcriptional activity to ADAM9 KD in HCT116 cells.

### KD of ADAM9 reduces Akt activity in both HCT116 and SW620 cells but inhibits downstream mTOR signaling in HCT116 only

**Figure 4:**
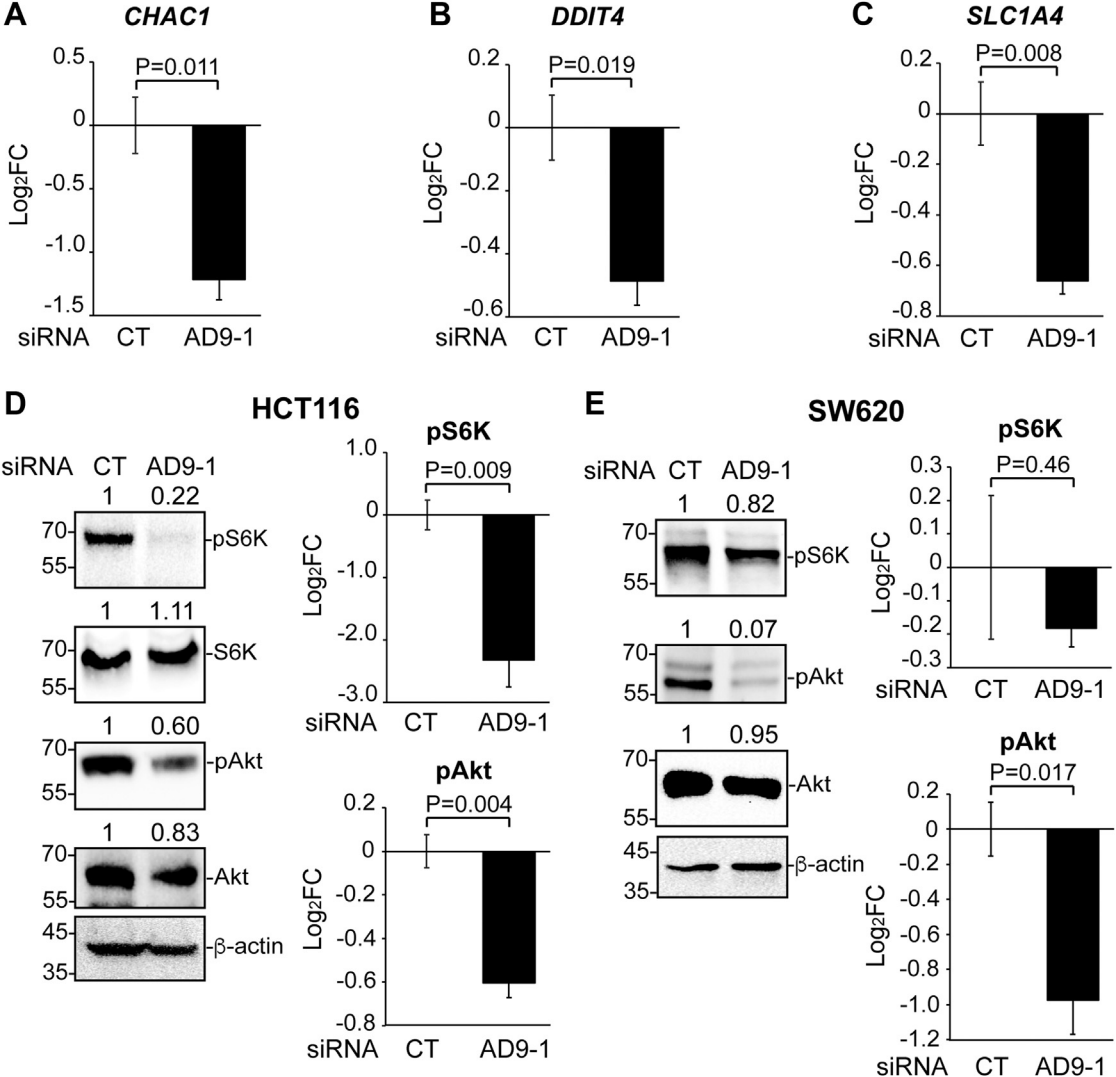
**KD of ADAM9 inhibits Akt activity but differentially affects mTOR signaling in HCT116 and SW620 cells.***A-C*, HCT116 cells were transfected with control or AD9-1 siRNA, and RT-qPCR was carried out for the indicated genes in three biological replicates. *D* and *E*, Western blotting was carried out for cell lysates with the antibodies for the indicated proteins in HCT116 (*D*) or SW620 (*E*) cells transfected with control or AD9-1 siRNA. Western blotting was repeated in triplicate to confirm the effects of ADAM9 KD on phospho-S6K and phosphor-Ser473 Akt (see Fig. S4 for images of blots); unpaired *t* tests were performed for log_2_FC of protein levels and summarized in graphs (error bars represent SEM). ADAM, A Disintegrin and Metalloprotease; KD, knockdown; RT-qPCR, quantitative RT-PCR.

If KD of ADAM9 has no effect on Wnt signaling in HCT116 cells, how does it inhibit the migration and invasion of these cells? To answer this question, we carried out RNA-seq analyses for HCT116 cells with control siRNA or siAD9-1. Among the 257 genes downregulated by siAD9-1, 31 were reported previously to be downregulated by mTOR inhibition (31), suggesting that KD of ADAM9 inhibits mTOR signaling (the complete RNA-seq data will be published elsewhere). RT-qPCR results confirm the reduced expression of *CHAC1*, *DDIT4*, and *SLC1A4*, three of the genes positively regulated by mTOR signaling (31), upon ADAM9 KD (Fig. 4, *A*–*C*). In line with this, KD of ADAM9 in HCT116 cells drastically reduced phospho-S6K, a signature mTOR target that facilitates tumor invasion including CRC invasion (32, 33, 34), without affecting total S6K levels (Fig. 4*D* and S4*A*). A main upstream regulator of mTOR signaling is Akt, which activates mTOR primarily by phosphorylating and inhibiting tuberous sclerosis complex 2 (TSC2), a well-characterized mTOR inhibitor (35, 36). Importantly, Akt can also promote Wnt signaling through various mechanisms (37, 38, 39). We therefore tested if ADAM9 regulates Akt activity. Indeed, transfection of HCT116 and SW620 cells with siAD9-1 reduced active (phospho-Ser473) Akt, while having little effect on total Akt (Fig. 4, *D* and *E*, S4, *B* and *D*). The inhibition of Akt activation by ADAM9 KD was confirmed using siAD9-2 (Fig. S5), suggesting that ADAM9 is required for Akt activation. However, the reduction in phospho-S6K was not significant in SW620 cells (Fig. 4*E* and S4*C*). Thus, while ADAM9 is indispensable for Akt activation in both cell lines, it appears to regulate the downstream Wnt and mTOR signaling differently in these cells. This phenomenon could be attributed to the underlying genetic mutations in these cell lines (see Discussion).

### ADAM9 function in CRC cell migration and invasion depends on Akt activation, possibly *via* the ephrin-B–PP2A axis

**Figure 5:**
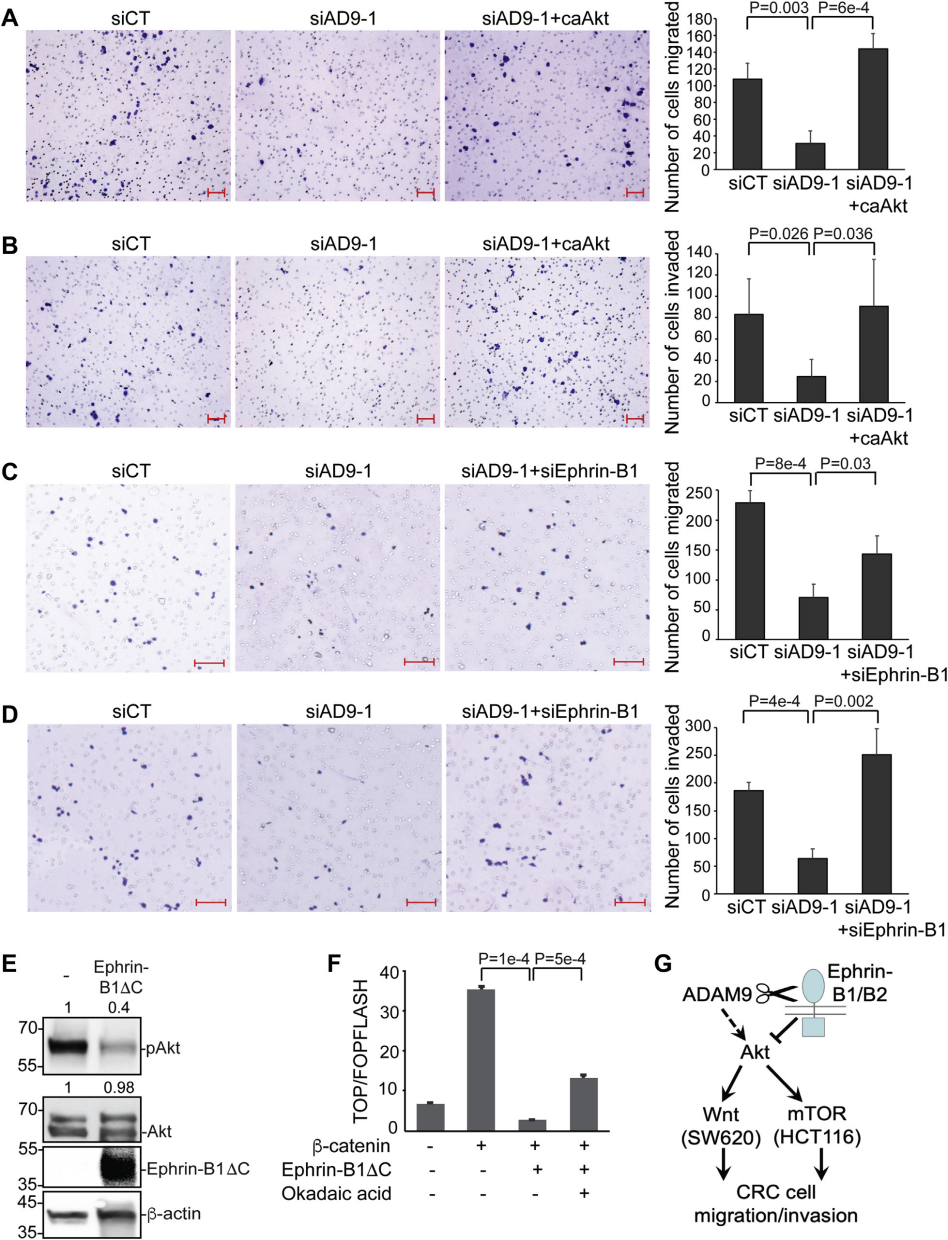
**ADAM9 activates Akt, possibly *via* the ephrin-B–PP2A axis, to promote CRC cell migration and invasion.***A*-*D*, SW620 cells were transfected with the indicated siRNA and plasmid, and transwell migration (*A* and *C*) and invasion (*B* and *D*) assays were performed in triplicate. *E*, HCT116 cells were transfected with empty vector or a plasmid encoding HA-tagged ephrin-B1ΔC, and Western blotting was carried out using the indicated antibodies (anti-HA for ephrin-B1ΔC). *F*, HEK293T cells were transfected with the indicated plasmids and treated with 1 μM okadaic acid, and TOP/FOPFLASH assays were carried out for cell lysates obtained for three biological replicates. *G*, a model for ADAM9 function in CRC migration and invasion. Unpaired *t* tests were performed in *A-D* and *F*; error bars represent SD. The scale bars represent 100 μm. ADAM, A Disintegrin and Metalloprotease; CRC, colorectal cancer; PP2A, protein phosphatase 2A.

We next tested if ADAM9 regulates CRC migration/invasion *via* ephrin-B signaling and Akt. Because SW620 cells are more migratory and invasive than HCT116 cells, we carried out loss- and gain-of-function experiments for ADAM9 in SW620 and HCT116 cells, respectively. As shown in Figure. 5, *A* and *B*, expression of a constitutively active form of Akt (40) rescued the reduced migration and invasion of SW620 cells caused by ADAM9 KD. As expected, the migration and invasion of HCT116 cells were enhanced by overexpressed ADAM9, and these effects were reversed by treating the cells with MK-2206, a selective Akt inhibitor (41)(Fig. S6). To test if ADAM9 functions in CRC cell migration/invasion by downregulating ephrin-B signaling, we used siRNAs that effectively knocked down ephrin-B1 (Fig. S7). Transfection of SW620 cells with these siRNAs rescued ADAM9 KD-mediated reduction of migration and invasion (Fig. 5, *C* and *D*). Together, these results suggest that ADAM9 promotes CRC cell migration and invasion by modulating ephrin-B signaling and Akt activity.

Finally, we asked how ADAM9 activates Akt. Akt is usually activated by PI3K, which can in turn be induced by growth factor–activated RTKs such as the epidermal growth factor receptor (EGFR) (35). A previous study has associated ADAM9 function in Akt regulation with EGFR activation (42). However, HCT116 cells contain a constitutively active PI3K mutation (Table S1), rendering Akt activity in these cells insensitive to growth factor stimulation (43), and a recent report shows that ADAM9 activates Akt independently of EGFR (44). We have shown that KD of ADAM9 results in increased levels of intact ephrin-B1 and active EphB3 receptor (Figs. 1, *E*–*G* and S1). Forward ephrin signaling can suppress Akt activity, even in cancer cells with oncogenic mutations that activate the PI3K–Akt axis (3). In particular, forward ephrin-B signaling *via* EphB3 can promote Akt dephosphorylation through protein phosphatase 2A (PP2A), leading to inhibition of lung cancer invasion (45). We therefore hypothesize that enhanced forward ephrin-B signaling mediates the inhibition of Akt and downstream signaling pathways upon ADAM9 KD. Consistent with this hypothesis, overexpression of an ephrin-B1 mutant with the cytoplasmic tail deleted (ephrin-B1ΔC), which can activate forward but not reverse signaling, inhibited Akt activity in HCT116 and SW620 cells, as well as ectopic β-catenin–induced Wnt signaling in HEK293T cells (Fig. 5, *E* and *F* and S8). Conversely, a dominant-negative EphB1 receptor with the kinase domain deleted (EphB1ΔK) enhanced AKT and Wnt activity in SW620 cells (Fig. S9, *A* and *B*). Of note, the inhibition of Wnt signaling by ephrin-B1ΔC in HEK293T cells can be partially rescued by the PP2A inhibitor okadaic acid (Fig. 5*F*), suggesting that forward ephrin-B signaling antagonizes Wnt signaling through PP2A.

## Discussion

ADAM9 is a tumor-associated antigen highly expressed in many types of solid tumors and is involved in carcinogenesis and tumor progression, but the underlying mechanisms remain poorly understood (26, 46). We found that *ADAM9* mRNA is also elevated in CRC tissues and identified Akt and downstream Wnt and mTOR signaling as the main targets for ADAM9 in CRC cells. Two published works have linked ADAM9 to Akt activity in esophageal squamous cell carcinoma and triple-negative breast cancer, respectively (42, 44). Together with our results, these suggest that ADAM9 is a key Akt regulator in various tumors. While the first study attributed this function of ADAM9 to EGFR activation, presumably by shedding the EGFR ligand pro-heparin binding-EGF (pro-HB-EGF) (42), the second one did not find any alterations in phospho-EGFR or the downstream phospho-MAPK upon ADAM9 KD (44). To resolve this contradiction, we used HCT116 cells, in which Akt activity is insensitive to growth factor stimulation due to the presence of a constitutively active PI3K mutation (43) and still observed a clear inhibition of the Akt–mTOR signaling axis caused by ADAM9 KD (Fig. 4, *D* and *E*). Moreover, although an earlier report implicated overexpressed ADAM9 in shedding of pro-HB-EGF (47), loss-of-function data do not support an essential role for endogenous ADAM9 alone in shedding pro-HB-EGF or any other EGFR ligands (44, 48, 49). Therefore, the effects of ADAM9 KD on EGFR activation observed previously are likely specific to the esophageal squamous cell carcinoma cells examined, and there are alternative mechanisms through which ADAM9 regulates Akt in other cells and tissues. As reported in lung cancer cells (45), we found that forward ephrin-B signaling suppresses Akt activity in CRC cells, possibly through PP2A-mediated Akt dephosphorylation (Fig. 5, *E* and *F*, S8 and S9). Because ADAM9 cleaves ephrin-B1 and -B2 and downregulates forward ephrin-B signaling (Fig. 1, *A*–*G*), we propose that ADAM9-mediated ephrin-B shedding activates Akt, as well as downstream Wnt and mTOR signaling in SW620 and HCT116 cells, respectively, to promote migration and invasion (Fig. 5*G*). In CRC, forward ephrin-B signaling is a tumor suppressor pathway, whereas Wnt and mTOR are two oncogenic pathways (5, 50, 51). Our results provide a mechanistic connection between ADAM9, which is highly expressed in CRC samples, and these tumor-related pathways.

Perhaps the most puzzling result of this study is the differential regulation of Wnt and mTOR signaling, two signaling pathways downstream of Akt, by ADAM9 in SW620 and HCT116 cells (Figs. 3 and 4). Akt can phosphorylate GSK3 to inhibit its activity (52), and this inhibition has been shown to cause β-catenin accumulation and Wnt activation in some cells (37, 53, 54). Additionally, direct phosphorylation of β-catenin by Akt at Ser552 can also lead to the dissociation of β-catenin from cell-cell contacts and enhanced Wnt reporter activity (38). In agreement with the latter mechanism, the PI3K pathway was found to primarily upregulate active β-catenin (39), which likely represents the transcriptionally active pool of β-catenin in the nucleus (30). However, other studies argue against an effect of Akt on Wnt signaling (55, 56), raising the possibility that the Akt-Wnt crosstalk is cell type specific (57). In line with this possibility, we found that KD of ADAM9 inhibits Wnt signaling in SW620 but not HCT116 cells. Biallelic inactivation of *APC* and gain-of-function mutations of *CTNNB1* are two most common types of mutations in CRC (58, 59). SW620 cells, along with SW480 cells derived from the same patient, contain a biallelic APC truncation that results in elevated Wnt activities (Table S1) (60, 61, 62). However, the truncated APC has been shown to retain partial function in forming the β-catenin destruction complex and regulating Wnt signaling (63, 64). Hence, it is not surprising that β-catenin levels and Wnt activity can still be reduced by ADAM9 KD in SW620 cells (Fig. 3, *B* and *D*). In contrast, HCT116 cells harbor a heterozygous 3-bp deletion in *CTNNB1* that eliminates Ser45 of the encoded β-catenin protein (Table S1) (62), a residue phosphorylated by CK1α as a prerequisite to prime the subsequent GSK3 phosphorylation and ubiquitination (65). This mutant allele contributes to nearly all the Wnt activities in HCT116 cells (66). However, the Ser45 deletion does not seem to affect β-catenin stability; instead, it decreases the binding affinity for cell-surface E-cadherin, resulting in redistribution of β-catenin from the plasma membrane to the cytoplasm and nucleus (66). Interestingly, KD of ADAM9 in HCT116 cells reduces total but not active β-catenin (Figs. 3*E* and S3*B*), suggesting that ADAM9-regulated Akt fails to affect β-catenin localization in this cell line. One possible explanation is that, in the absence of Ser45 phosphorylation, β-catenin cannot localize to the plasma membrane due to the low affinity for E-cadherin and probably other classical cadherins, even when Akt activity and Ser552 phosphorylation are reduced by ADAM9 KD. Since LEF/TCF-mediated transcriptional activity correlates with active but not total β-catenin (30), the decreased total β-catenin is not sufficient to influence target gene expression (Fig. 3*C*).

Although KD of ADAM9 in HCT116 cells greatly inhibits S6K phosphorylation, a hallmark of mTOR signaling, we did not observe a comparable effect in SW620 cells (Fig. 4, *D* and *E*). Besides elevated Wnt signaling, oncogenic APC inactivation also leads to hyperactivation of mTORC1, and inhibition of mTORC1 protects from intestinal tumorigenesis and certain other defects caused by APC inactivation (67, 68, 69). The antagonism of mTORC1 by APC has been shown to depend on GSK3, which phosphorylates TSC2; importantly, phosphorylation by GSK3 is required for TSC2’s ability to inhibit mTORC1 signaling (70, 71). Of note, the primary mechanism through which Akt activates mTORC1 is by phosphorylating and inhibiting TSC2 (35). While the relationship between these two phosphorylation events remains unclear, it is possible that APC mutations, such as the one found in SW620/480 cells, result in loss of GSK3-mediated TSC2 phosphorylation and abolished TSC2 function, which cannot be restored by reduced Akt activity. We also cannot rule out any possible contribution from other mutations in SW620 cells, such as the mutations in *P53* (Table S1) (72), as cross-inhibition of mTOR by p53 has been demonstrated previously (73).

Akt is an important therapeutic target for multiple types of cancers including CRC, and Akt inhibitors have been actively developed and tested for cancer treatment (74, 75). However, there remain some challenges that need to be overcome. As a signaling node, Akt is the converging point of multiple upstream pathways and regulates various downstream outputs (35, 36). In light of the recent failure of Akt inhibitors in clinical trials, it is important to better understand the upstream regulators and downstream effectors of this signaling node and to select patients that may respond well to treatments based on their genetic backgrounds (76). We show here that ADAM9 functions as a key upstream regulator of Akt in CRC cells, possibly by cleaving class B ephrins, and that KD of ADAM9 differentially regulates downstream Wnt and mTOR signaling in CRC cell lines with different genetic mutations. These results point to the feasibility of targeting ADAM9 as a potential means to modulate Akt activity and using patients’ genetic backgrounds to predict the outcomes of these manipulations.

## Experimental procedures

### Plasmids and reagents

The expression construct for caAKT was a gift from Dr Richard Roth (Addgene #10841) (40). The complementary DNAs (cDNAs) for mouse EfnB1 (encoding ephrin-B1), EfnB2 (encoding ephrin-B2), and EfnB1ΔC were subcloned into a pCS2+ expression vector with an in-frame N-terminal HA tag inserted a few residues after the predicted signal peptide cleavage site, and C-terminally myc_6_-tagged WT ADAM9 and ADAM9(E348A) were subcloned into pCS2+ (see Table S2 for primer sequences). The *Xenopus laevis* EphB1ΔK construct was a gift from Dr Ira Daar. MK-2206 was purchased from Selleckchem (Cat. #: S1078). Antibodies used in this study include rabbit anti-HA (Cell Signaling Technology (CST) 3724, 1:1000), mouse anti-myc (CST 2276, 1:2000), rabbit anti-ADAM9 (CST 4151, 1:1000), rabbit anti-phospho-EphB3(Y608) (Invitrogen PA564791, 1:500), rabbit anti-active-β-catenin (CST 8814, 1:1000), rabbit anti-β-catenin (CST 9582, 1:1000), rabbit anti-phospho-p70 S6 kinase (CST 97596, 1:1000), rabbit anti-p70 S6 kinase (CST 2708, 1:1000), rabbit anti-phospho-AKT(S473) (CST 4060, 1:1000), and rabbit anti-AKT1/2/3 (Abcam 179463, 1:10,000). Secondary antibodies that were used include horseradish peroxidase (HRP)-conjugated rabbit anti-mouse (CST 7076, 1:7500) and goat anti-rabbit (CST 7074s, 1:5000). HRP-conjugated mouse anti-β-actin (CST 12262, 1:10,000) was used as a loading control. The following siRNAs were used in this study: control (CST 6568), AD9-1 (CST 11968), AD9-2 (Ambion 4390826), and ephrin-B1 (Dharmacon L-003658-00-0010).

### Cell culture and transfection

HCT116 cells (ATCC) were cultured in McCoy’s 5A (ATCC) supplemented with 10% fetal bovine serum (FBS, Gibco) at 37 °C with 5% CO_2_. SW620 cells (ATCC) were cultured in Leibovitz's L-15 (ATCC) and supplemented with 10% FBS at 37 °C with 0% CO_2_. Cells were transfected with plasmids (400 ng/ml) or siRNA (100 nM) at 70% confluency, using Lipofectamine 3000 (Invitrogen). For experiments that used both siRNA and plasmid, siRNA was transfected first, and the medium was replaced with fresh before the plasmid was transfected on the following day. For TOP/FOPFLASH assays, cells were transfected with TOPFLASH or FOPFLASH construct for 24 h and luciferase assays were carried out as described previously (18). For cleavage assays, cells were transfected and subsequently cultured in serum-free medium for 48 h (18), and Western blotting was performed as described below.

### Western blotting and RT-qPCR for cell lysates

To prepare whole-cell lysates, cells were lysed in ice-cold RIPA buffer (Invitrogen) supplemented with protease and phosphatase inhibitor cocktails (Invitrogen) and o-phenanthroline (Millipore Sigma) at 10 mM final concentration for 30 min on ice and centrifuged at 18,000*g* for 10 min at 4 °C to remove any debris. The amount of protein in each sample was measured by bicinchoninic acid assay (Millipore Sigma), and 30 μg protein extract was loaded per lane for Western blot analyses. For cleavage assays, the conditioned media were concentrated by centrifugation at 6000*g* for 30 min at 4 °C using centrifugal filters with 3 kDa molecular weight cutoff (Millipore UFC500396), and 40 μl of the supernatant was loaded per lane for Western blot analyses. SDS-PAGE was run on precast acrylamide gels (Bio-Rad) until the desired separation was achieved, and gels were transferred onto 0.2 um pore PVDF membranes (Bio-Rad) using the Trans-Blot Turbo transfer system (Bio-Rad). Western blot detection was conducted with HRP-conjugated secondary antibodies and chemiluminescence substrates (Bio-Rad) using a Bio-Rad ChemiDoc imager, as described previously (21). For RT-qPCR, total RNA was isolated using the RNeasy Mini Kit (Qiagen) according to the manufacturer's instructions. RNA quality was assessed by electrophoresis on 1% agarose gels, and RNA quantity was determined by NanoDrop One Spectrophotometer (NanoDrop Technologies). Total RNA was reverse transcribed into cDNA using the iScript cDNA Synthesis Kit (Bio-Rad Laboratories) with DNase I treatment (Qiagen). Quantitative PCR was performed with a Quant Studio 6 Flex (Applied Biosystems) using the qMAX Green Low Rox qPCR Mix (Accuris) and corresponding primers (see Table S2 for sequences). Relative quantity was calculated by normalizing to the amount of *GAPDH* mRNA.

### Transwell migration and invasion assays

For transwell migration assays, 5 × 10^4^ cells were plated in the top chamber of 8.0 μm inserts (Falcon 353097). For invasion assays, wells were coated with Matrigel according to manufacturer’s protocol (Corning 354234). Cells in serum-free medium were plated in the upper chamber, and the medium containing 10% FBS was added in the lower chamber. After 22 h of incubation at 37 °C, the cells were fixed in 4% formaldehyde and stained with 0.1% crystal violet, and cells that invaded through the pores were counted under a Zeiss Axio V16 microscope. Three chambers were used per condition. The values obtained were calculated by averaging the total number of cells from three filters.

### Animals and embryo manipulation

Male and female *X. tropicalis* adults were purchased from NASCO. Live animal manipulation was performed in accordance with the guidelines and regulations set and enforced by the Institutional Animal Care and Use Committees at West Virginia University and the University of Delaware. Morpholino oligo targeting ADAM13 (13-3) was designed and generated by Gene Tools (18). *In vitro* transcription, embryo manipulation, injection, and culturing were carried out as described previously (77). Briefly, two-cell stage embryos were injected in one blastomere with 6 ng morpholino 13-3 and 100 pg mouse ADAM9 mRNA using PLI-100A microinjectors (Harvard Apparatus). Embryos were collected at NF stage 12.5, fixed in 4% paraformaldehyde for 24 h at 4 °C, and *in situ* hybridization was conducted subsequently as described (78). Embryos were scored by comparing the injected side with the uninjected side of the same embryos. The percentage of embryos with normal and reduced *snai2* expression were calculated for three independent experiments, and Chi-squared tests were performed to compare the percentage of embryos with normal phenotypes in different treatment groups.

### Human CRC sample collection and RT-qPCR analyses

Forty-eight pairs of human CRC specimens and surrounding nontumor colon tissues were obtained from the Zhongshan Hospital of Xiamen University with patient consent and institutional review board approval. The protocol conformed to the ethical guidelines was approved by the Institute Research Ethics Committee at Xiamen University. Total RNA was isolated from human CRC specimens and surrounding nontumor colon tissues using Trizol reagent (Invitrogen). cDNA was obtained from 2 μg of total RNA using Revertra Ace qPCR RT master mix (TOYOBO). Quantitative real-time PCR was performed using Faststart universal SYBR green master (Roche), and relative quantification was calculated by normalizing to the amount of β-actin mRNA. See Table S2 for primer sequences.

## Data availability

All data are contained within this article.

## Supporting information

This article contains supporting information.

## Conflict of interest

The authors declare that they have no conflicts of interest with the contents of this article.

## Supporting information

Supporting information revised
