# Supplementary material for: Metalloprotease ADAM9 cleaves ephrin-B ligands and differentially regulates Wnt and mTOR signaling downstream of Akt kinase in colorectal cancer cells
Source: J Biol Chem. 2022 Jul 1;298(8):102225. doi: 10.1016/j.jbc.2022.102225 (PMC9358476; doi:10.1016/j.jbc.2022.102225)
Supplement: Supporting information revised [file mmc1.pdf]

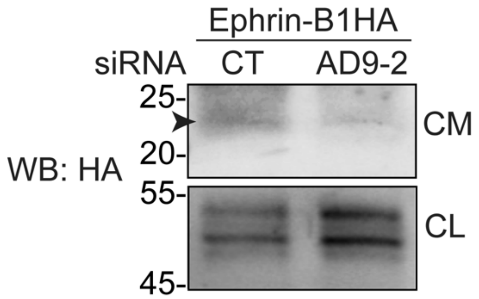

Figure S1 Transfection of HCT116 cells with siAD9-2 inhibits ephrin-B1 shedding. HCT116 cells were transfected with a plasmid encoding N-terminally HA-tagged ephrin-B1 with siControl (CT) or siAD9-2, and western blotting was carried out for conditioned media (CM) or cell lysates (CL) using an anti-HA antibody.

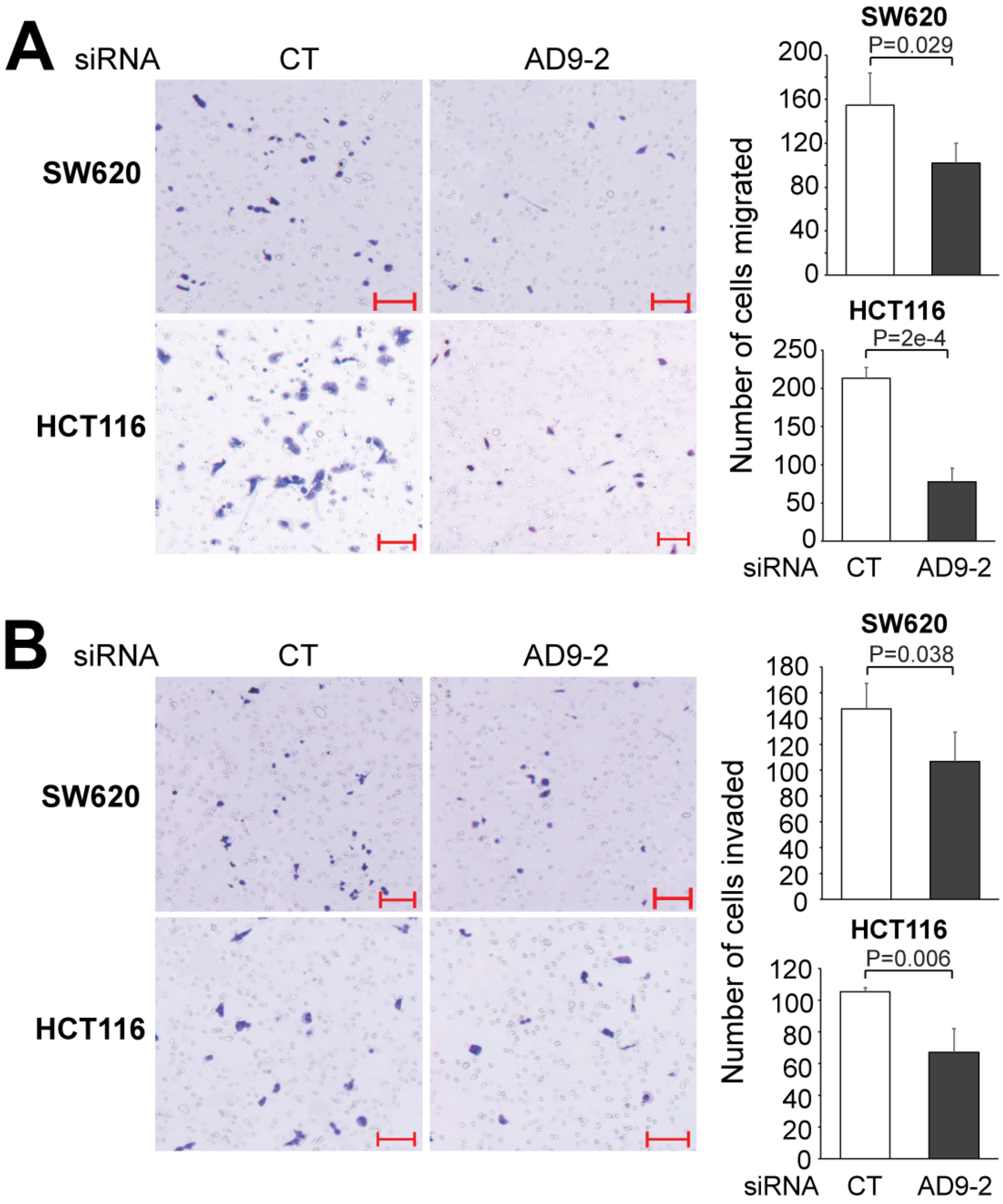

Figure S2 Transfection of SW620 and HCT116 cells with siAD9-2 inhibits migration and invasion. SW620 or HCT116 cells were transfected with the indicated siRNA, and transwell migration (*A*) and invasion (*B*) assays were carried out as described in *Experimental procedures*. Results of 3 biological replicates for each treatment are summarized on the right, and unpaired *t* test was performed. Error bars represent standard deviation. Scale bars, 100 μm.

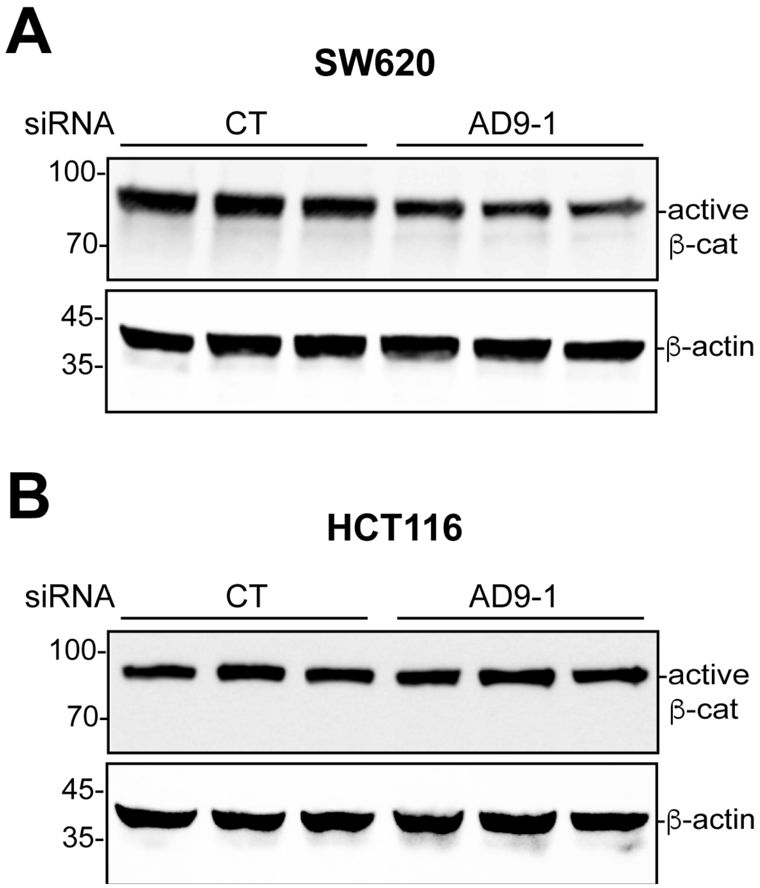

Figure S3 KD of ADAM9 reduces active β-catenin in SW620 but not HCT116 cells. SW620 (*A*) and HCT116 (*B*) cells were transfected with the indicated siRNA, and western blotting was performed for cell lysates using the indicated antibodies. Experiments were carried out in triplicate, and statistics are presented in Fig. 3D and E, respectively.

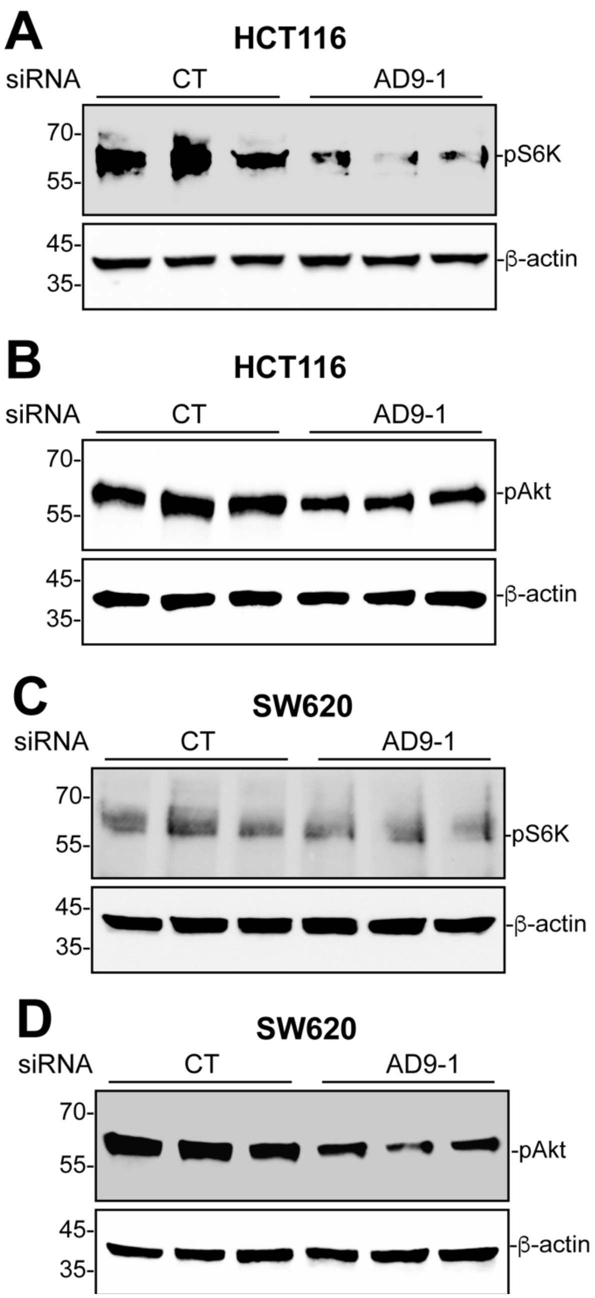

Figure S4 Effects of ADAM9 KD on phospho-S6K and phospho-Ser473 Akt in HCT116 and SW620 cells. HCT116 (*A* and *B*) and SW620 (*C* and *D*) cells were transfected with the indicated siRNA, and western blotting was performed for cell lysates using the indicated antibodies. Experiments were carried out in triplicate, and statistics are presented in Fig. 4D and E, respectively.

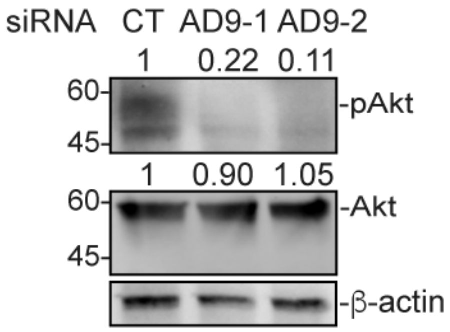

Figure S5 Transfection of HCT116 cells with either siAD9-1 or siAD9-2 inhibits Akt activity. HCT116 cells were transfected with the indicated siRNA, and western blotting was carried out for cell lysates using the indicated antibodies.

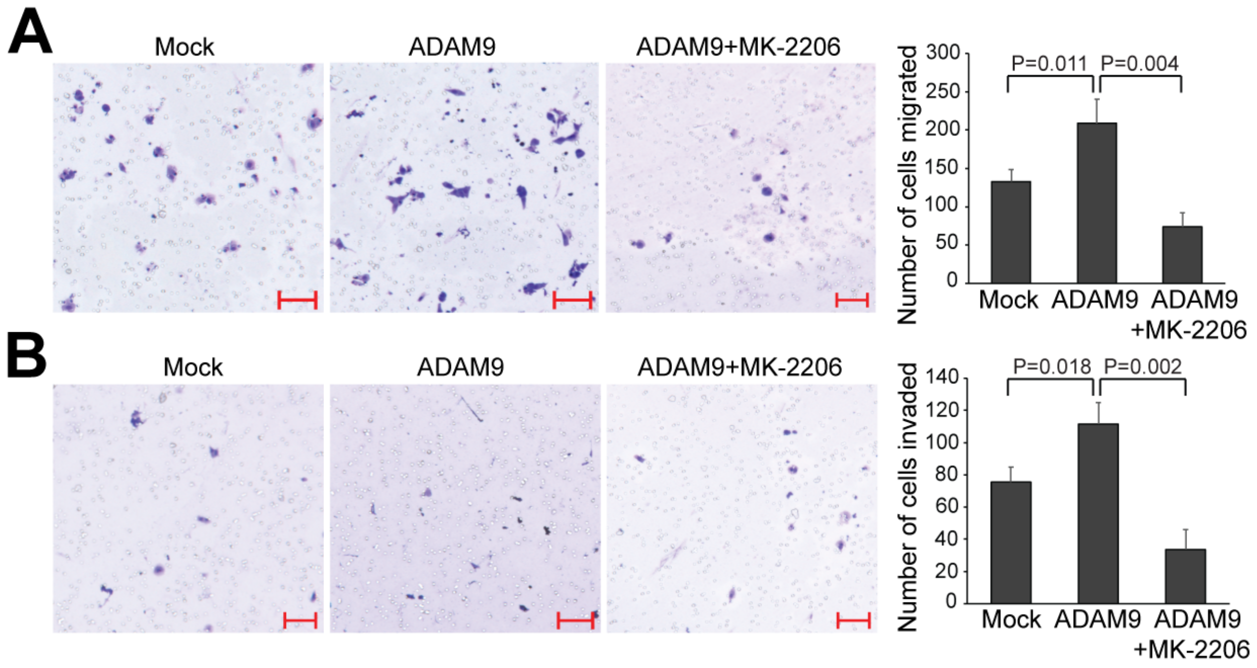

Figure S6 Overexpression of ADAM9 in HCT116 cells leads to increased migration and invasion, which can be blocked by Akt inhibition. HCT116 cells were transfected with empty pCS2+ vector (Mock) or pCS2+:*Adam9*, and treated with DMSO (vehicle control) or 12.5 nM of the selective Akt inhibitor MK-2206. Transwell migration (*A*) and invasion (*B*) assays were carried out. Results of 3 biological replicates for each treatment are summarized on the right, and unpaired *t* test was performed. Error bars represent standard deviation. Scale bars, 100 µm.

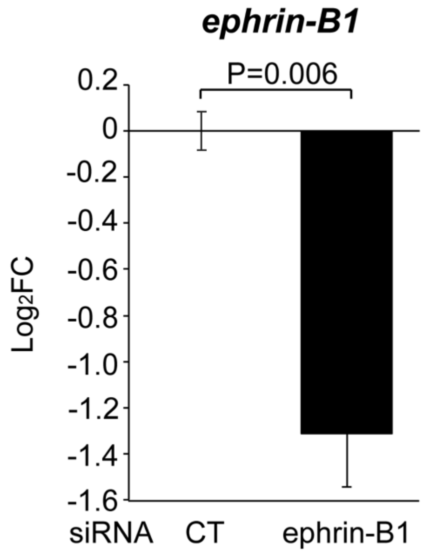

Figure S7 The ephrin-B1 siRNAs effectively reduce ephrin-B1 mRNA in SW620 cells.

SW620 cells were transfected with the indicated siRNAs, and RT-qPCR was carried out for *ephrin-B1* mRNA. Unpaired *t* tests were performed for $\log_2$FC of mRNA levels obtained for 3 biological replicates, and error bars represent standard error of the mean (SEM).

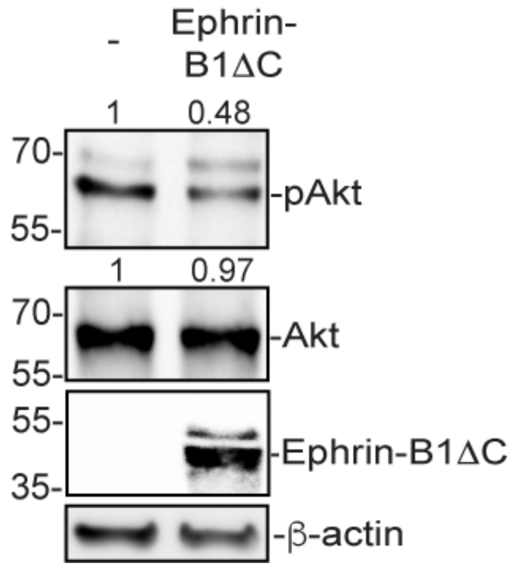

Figure S8 Overexpression of ephrin-B1ΔC in SW620 cells inhibits Akt activity. SW620 cells were transfected with an empty vector or a plasmid encoding HA-tagged ephrin-B1ΔC, and western blotting was carried out for cell lysates using the indicated antibodies (anti-HA for ephrin-B1ΔC).

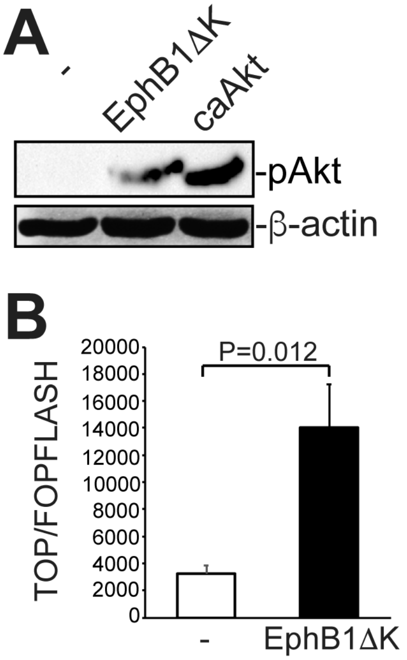

Figure S9 Overexpression of EphB1ΔK in SW620 cells enhances Akt and Wnt activities. SW620 cells were transfected with an empty vector or a plasmid encoding HA-tagged EphB1ΔK, and western blotting (*A*) or TOP/FOPFLASH luciferase activity assay (*B*) was carried out for cell lysates. Cells transfected with a plasmid encoding caAkt were used as a positive control in *A*. Unpaired *t* test was performed to compare Wnt activity in B, and error bars represent standard deviation.

Table S1 Comparison of key mutations in HCT116 and SW620 cells

	HCT116	SW620
**K-Ras**	G13D	G12V
**B-RAF**	WT	WT
**PTEN**	WT	WT
**PIK3CA**	H1047R	WT
**APC**	WT	Q1338Ter
**CTNNB1**	Ser45del	WT
**P53**	WT	R273H; P309S

Table S2 PCR primers used in this study

Name	Sequence (5' to 3')
mADAM9-BamH1-forward	ACCTGCGGATCCCTCGCTATGGGGCCG
mADAM9-Cla1-reverse	GCGCATCGATGGGGTGAGGGAGCTATATAA
mADAM9-E348A-reverse	ATTATGACCCAATGCATGAGCAACAAT
mADAM9-E348A-forward	ATTGTTGCTCATGCATTGGGTCATAAT
mEFNB1-HA-forward	TACCCATACGATGTTCCAGATTACGCTCTGGAGCCCGTGTCCTGG
mEFNB1-HA-reverse	AGCGTAATCTGGAACATCGTATGGGTAGTTCTTGGCCAACGGCGT
mEFNB1-ClaI-forward	TGCATCGATGAAAATGGCCCGGCC
mEFNB1-XhoI-reverse	GCTGCTCGAGAGTCAAACCTTGTAG
mEFNB1ΔC-XhoI-reverse	TTCACTCGAGGAGTAGTAGGACTGTCAAGAAGATG
mEFNB2-HA-forward	TACCCATACGATGTTCCAGATTACGCTTTAGAGCCTATCTACTGG
mEFNB2-HA-reverse	AGCGTAATCTGGAACATCGTATGGGTAAACTATCGATCTGGAGAT
mEFNB2-BamH1-forward	GAGCGGATCCGGCATGGCCATGGCCCGG
mEFNB2-Xho1-reverse	GCCGCGCTCGAGAGTCAGACCTTGTAGTAAATG
hADAM9-forward for RT-qPCR	CTTGCTGCGAAGGAAGTACC
hADAM9-reverse for RT-qPCR	AACATCTGGCTGACAGAACTG
hβ-actin-forward for RT-qPCR	AGCGAGCATATCCCCCAAAGTT
hβ-actin-reverse for RT-qPCR	GGGCACGAAGGCTCATCATT
hCHAC1-forward for RT-qPCR	GTGGTGACGCTCCTTGAAGATC
hCHAC1-reverse for RT-qPCR	GAAGGTGACCTCCTTGGTATCG
hDDIT4-forward for RT-qPCR	GTTTGACCGCTCCACGAGCCT
hDDIT4-reverse for RT-qPCR	GCACACAAGTGTTCATCCTCAGG
hSLC1A4-forward for RT-qPCR	CCTCACCATTGCCATTATCTT
hSLC1A4-reverse for RT-qPCR	CATCCCCTTCCACATTCACC
hGAPDH-forward for RT-qPCR	TCAAGGCTGAGAACGGGAAG
hGAPDH-reverse for RT-qPCR	CGCCCCACTTGATTTTGGAG
hEFNB1-forward for RT-qPCR	GTCCTACTACTGAAGCTACG

hEFNB1-reverse for RT-qPCR	CTCTTGGACGATGTAGACAG

Note: Restriction sites introduced are underscored.
